# Hydroethanolic Extracts of* Erigeron floribundus* and* Azadirachta indica* Reduced* Plasmodium berghei* Parasitemia in Balb/c Mice

**DOI:** 10.1155/2018/5156710

**Published:** 2018-10-21

**Authors:** Roselyne Nzangue Tepongning, Javeres Ntepe Mbah, Francky Love Avoulou, Marie Madeleine Jerme, Evrard-Kevin Kene Ndanga, Fabrice Boyom Fekam

**Affiliations:** ^1^Department of Biomedical Science, University of Ngaoundere, P.O. Box 454, Ngaoundere, Cameroon; ^2^Antimicrobial & Biocontrol Agents Unit, Department of Biochemistry, University of Yaounde I, P.O. Box 812, Yaounde, Cameroon

## Abstract

Malaria is one of the most important infectious diseases in Africa especially in Cameroon. The nonaccessibility to current treatments for poor people and the appearance of drug-resistant* Plasmodium falciparum* parasites stimulate the search for alternative treatments. The aim of this study was to evaluate the antimalarial activity and the safety of hydroethanolic extracts from* Erigeron floribundus* and* Azadirachta indica*. The crude hydroethanolic extracts of* E. floribundus* (HEEF) and* A. indica* (HEAI) were prepared via maceration of the whole plant powder of* E. floribundus* and the leaves of* A. indica* in 70% ethanol. The antimalarial activity was determined according to Peter's 4-day suppressive test using the murine model* Plasmodium berghei*/*Balb C* mice, while the acute and subacute toxicity tests were assessed according to the OECD 425 and 407 guidelines, respectively. The results indicate a reduction of parasitemia ranging from 49.75 ± 3.64 to 69.28 ± 1.36% for HEAI and from 30.46 ± 4.30 to 62.36 ± 2.32% for HEEI. Overall, HEEF and HEAI at doses of 60, 120, and 240 mg/kg b.w. and 75, 150, and 300 mg/kg b.w., respectively, showed a significant (*p*≤0.001) parasitemia reduction on* P. berghei* infecting BALB/c mice. HEEF and HEAI caused a significant (*p*<0.001) attenuation of body temperature drop in mice compared to negative control, except for the 150 mg/kg b.w. dose in the female group. Moreover, there was no mice mortality observed with these extracts even at 5000 mg/kg, while the aspartate amino transferase (ASAT) level of mice treated with 300 mg/kg b.w. of HEAI extract increased when compared with the control. The results of this study support the traditional use of these plants species extracts against malaria infection in rural zones of Northern Cameroon, therefore confirming their potential as sources for the development of efficient phytomedicines for malaria-poverty disease alleviation.

## 1. Introduction

Malaria transmitted by a mosquito remains a public health problem with around 88% diagnosed cases in sub-Saharan Africa. Cameroon is one of the most affected countries in Africa with around 4500 deaths recorded annually, particularly in the Northern region [1]. The strategies applied to eradicate this infection include chemotherapy consisting of antimalarial drugs. However, the recent development of drug-resistant Plasmodium species [2] and the difficulties of infected populations to access the treatments have stimulated the search for alternatives medicines.

Ethnobotanical surveys have been conducted in several regions of Africa and Cameroon, revealing more than 40 medicinal plants species belonging to more than 30 families used in the treatment of malaria [3–7]. A majority of the African population still relies on the use of traditional herbal medicines to treat malaria especially that of Northern Cameroon, influenced by ancestral and religious beliefs, who seldom visits hospitals. Different parts of plants species including leaves, stem barks and roots are frequently used to prepare infusion, concoction, or decoction for the prophylactic and treatment purposes [7], where they are administered orally, in bath or steam inhalation for 3 to 6 days, or more often until parasites clearance or not [8].

*Azadirachta indica* A. Juss is one of the medicinal plants widely used in the treatment of malaria and several diseases in Central and West Africa [9–11]. One well known formulation for infected patient consists of macerating the whole fruits in water for 2 hours, and drinking one-quarter glass three times a day for 5 to 7 days [3]. The traditional use of* Erigeron floribundus* (Kunth) Schultz-Bip. is not well documented, although ethnobotanical review revealed its antifungal [12], analgesic and anti-inflammatory [13], genital anti-infective [14], and antitussive [15] activities.

For their antiplasmodial potential, several studies reported the efficacy of these two plants extracts. For example, methanolic extracts of* A. indica* was reported to inhibit the growth of chloroquine sensitive strain 3D7 and chloroquine resistant strain Dd2 of* Plasmodium falciparum* with IC_50_ values of less than 5 *μ*g/mL [16], while fresh clinical isolates of* P. falciparum* were inhibited with IC_50_ values of 2 and 16,9 *μ*g/mL for aqueous and methanolic extracts, respectively [17]. Pentane and ethanol extracts of* E. floribundus* were found to inhibit the growth of* P. falciparum* with IC_50_ values ranging from 15-35 *μ*g/mL [18]. Also, the* E. floribundus* aqueous extracts were found to inhibit* P. falciparum* amodiaquine-sensitive and resistant isolates, with IC_50_ values range 6.78±1.97 to 26.17 *μ*g/mL [19].

Antimalarial activity studies conducted with* A. indica* derived from the fruit and leaves have evidenced interesting results. Indeed, a prophylactic activity of ethanolic extract of neem fruit was exhibited on BALB/c mice infected with* Plasmodium berghei* ANKA strain with 43 to 47 % reduction of parasitaemia with respect to controls [20]. Similarly, aqueous extracts from the leaves of* A. indica* showed parasitemia percent chemosuppression of 48.95±17.58 % and 62.50±2.05 % at doses of 50 and 100 *μ*g/mL, respectively, in Ghana [21]. Although many studies have already reported antiplasmodial activity and toxicity of* A*.* indica*, no study has yet corroborated this activity for* A*.* indica* in Cameroon, yet it is a plant with a high consumed in the localities of the north Cameroon. The study of* A*.* indica* in Cameroon during our study thus finds all its relevance. Similarly and for the best of our knowledge, the antimalarial properties of* E. floribundus* have not been studied yet.

Given the popular use of* A. indica* and the traditional medicinal value attributed to a little-well known plant,* E. floribundus*, in the Dibi locality, information on their efficacy and safety is relevant to confirm their use. Our study aims at evaluating, comparing the antimalarial potential of the hydroethanolic extracts of the 2 plants using the murine malaria system* P*.* berghei/Anopheles stephensi* and BALB/c mice and then exploring their safety.

## 2. Materials and Methods

### 2.1. Plant Collection and Extracts Preparation

The plant samples were collected in the Adamawa Region (locality of Dibi) after interviews among traditional doctors who gave their consent to talk about malaria, from the 29 December 2014 to the 08 January 2015. Information gathered from traditional doctors was needful to identify the plants used in the selected area for the management of malaria. These plants were harvested according to interview details early in the morning before sunrise. They were later identified by a plant taxonomist and further confirmation was made at the National Herbarium in Yaounde, Cameroon. The questionnaire used during the interview included information on the local use, the local name, the mode of preparation, the forms of administration of the medicinal plants, the parts of the plants used, the dosage and duration of the treatment, and recipes for the management. Among these selected plants, two plants were chosen for this preliminary study:* E*.* floribundus* (scarce) and* A*.* indica* (abundant) and voucher specimens are deposited at the National Herbarium Cameroon with reference numbers of 11132/HNC and 4447/SRFK, respectively. The plant sampling was done in accordance with the Convention on the Trade in Endangered Species of Wild Fauna and Flora.

The entire plant of* E*.* floribundus* and leaves of* A*.* indica* were cut into small pieces, dried at laboratory temperature (25°C) for two weeks till constant weight and powdered. Powdered plant materials (100 g) were macerated in 1500 mL of ethanol/water (70:30) for 24 h at room temperature. The resulting extract was filtered and evaporated using a rotary evaporator at 40°C. The residues which constitute the crude extracts were recovered in flasks and then left in an oven at 40°C until complete evaporation of the solvent. All extracts were then kept at 4°C until further use. The different doses of plant extract were prepared using 100 mg/mL stock solutions.

### 2.2. The Animals

For* in vivo* antimalarial assay, 8-12 weeks old BALB/c mice (60 male and 60 female) weighing between 20 and 30 g were used. For toxicity test, 6 to 8 weeks old* Rattus norvegicus* rats (40 male) weighing between 100-180 g were used. The animals were obtained from the National Veterinary Laboratory (Garoua-Cameroon) and were kept in laboratory conditions for two weeks before the experiment. These conditions included: temperature 22°C (±3°), 12 h light/dark cycle, food and water provided* ad libitum*. All experiments were conducted according to the European Union guidelines for experimentation on laboratory animals (Directive 2010/63/EU of 8 August 2010 on the protection of animals used for scientific purposes).

### 2.3. *In Vivo* Antimalarial Activity of Extracts

The antimalarial potency of extracts was studied as described in the classical 4-day suppressive test of Peters [22]. Briefly, mice were inoculated intraperitoneally with 200 *μ*L of 1 x 10^7^* P*.* berghei *NK65 strain in blood. Three hours after, HEEF and HEAI extracts were administrated orally. The treatment was carried out for 4 days. Doses used were 60, 120 and 240 mg/kg for HEEF and 75, 150 and 300 mg/kg for HEAI. The group considered as negative control received 200 *μ*L of distilled water, while the group of positive control received 10 mg/kg of quinine.

Smears were prepared on the fifth day and the parasitemia was determined microscopically (magnification x100) using the following formula of Zucker and Campbell [23]:

(1) $$\begin{array}{c} \begin{array}{c} \%\;\;Parasitemia=\frac{Number\;\;of\;\;infected\;\;red\;\;blood\;\;cells}{Total\;\;number\;\;of\;\;red\;\;blood\;\;cells\;\;examined}\times 100 \end{array} \end{array}$$

The efficacy of the treatment for each extract was evaluated by calculating the average percent reduction of parasitemia according to equation 2:

(2) $$\begin{array}{c} \%\;\;reduction=\frac{C-T}{C}\times 100 \end{array}$$

C is mean parasitemia percentage in the negative control group and T is mean parasitemia percentage in the treated group.

During the 4-day suppressive test, the body temperature of each mouse was determined before infection on day 0 and after the treatment on day 4 using a rectal thermometer. The method involves inserting a small diameter temperature probe covered with Vaseline through the anus of the mouse. Before that the mouse is hand-restrained and placed on a horizontal surface and its tail lifted.

### 2.4. Evaluation of Acute Toxicity

The acute toxicity of HEEF and HEAI was evaluated in female mice according to the OECD 425 guidelines [24] for trials using chemical substances. The test substance was given sequentially in a single dose of 2000 mg/kg to a total of five mice, using a stomach tube. Later, the substance was administered sequentially at a dose of 5000 mg/kg to a single animal up to three animals at 48 hours interval. After administration, animals were observed individually at least once during the first 30 minutes after dosing; the operation was repeated periodically during the first 24 hours (with special attention given during the first 4 hours) and daily thereafter, for a total of 14 days. The animals weights were measured on days 0, 7, and 14 after administration then the survived animals were subjected to gross necropsy at the end of the test period to evaluate pathological changes.

### 2.5. Evaluation of Subacute Toxicity

The subacute toxicity profile of HEEF and HEAI was evaluated using male rats according to OECD 407 guidelines [25]. Briefly, for each group of 5 rats, oral doses of 60, 120, and 240 mg/kg for HEEF and 75, 150, and 300 mg/kg of HEAI were administrated daily for 28 days. The control group received the distilled water. The weight of each animal was recorded weekly during the experimental period and the signs of toxicity or mortality observed.

### 2.6. Serum Preparation and Macroscopic Analyses of Organs

Twenty-eight days after administration of extracts, animals were fasted overnight and sacrificed. Blood samples were collected by cardiac puncture and centrifuged at 3000 rpm for 5 min to obtain serum. The organs including the liver, the heart, the kidney, the spleen, and the lung were removed, observed, and weighed.

### 2.7. Measurement of Biochemical Parameters

At the end of the repeated dose 28-day oral toxicity study, blood collected from thiopental anesthetized rats by cardiac puncture was introduced in tube, swirled, and placed on ice. The serum was obtained by centrifugation at 3000 rpm for 5 min for biochemical measurements, using Biolabo commercial diagnosis kits for creatinine (80107), alanine aminotransferase (92027), and aspartate aminotransferase (92025).

### 2.8. Data Analysis

Quantitative data were expressed as arithmetic mean ± standard deviation (SD) of three to six replicates. To compare the parasitemia level, the independent samples Student's t-test was used, and one-way analysis of variance (ANOVA) followed by the Tukey HSD post hoc tests applied for the other results. Mean values were considered to be statistically significant at p<0.05.

## 3. Results

### 3.1. Plant Selection

At the end of the ethnobotanical survey in the locality of Dibi, Adamawa Region, 11 plants were recorded to be used for antimalarial cure (Supplementary Material 1). Plants were* A*.* indica*,* Tamarindus indica, Carica papaya*,* Senna occidentalis*,* Eucalyptus *sp.,* Bidens pilosa*,* Lantana camara*,* Achyranthes aspera*,* Spermacoce stachydea*,* Aloe vera,* and* E*.* floribundus*. Two plants (*A*.* indica* and* E*.* floribundus*), the most known/studied and the least cited, respectively, were selected for biological assessment.

### 3.2. Plant Preparation and Administration

The results of ethnobotanical study in Dibi locality revealed that maceration (45.5%) and decoction (72.7%) were the modes of preparation used by traditional doctors for malaria remedies (Supplementary Material 1). Fresh parts of the plant were generally used to treat malaria except for* A*.* indica*,* C*.* papaya*,* S*.* occidentalis,* and* Eucalyptus *sp. stored as dried powders in closed bottles. Most plants were administered orally (84.4%), soaked in hot or cold water, and taken orally as the active medicine. Only vapors of* A*.* indica* were inhaled during the treatment. The plant parts collected were leaves, roots, bark, and flowers/fruits. Remedies based on mixtures of different plants were also common.

The amounts of solution used for remedy recorded during the survey were described in terms of a full, half, or quarter of a cup of 250 mL to be taken two or three times a day for three to seven days or until the patient is healed. Different dosages for the same preparation were described by different healers. The most common usage was to keep the preparation in the jar of 500 mL, 1L, 1,5L, and 5L. No clear pattern or any consistency existed between healers as far as dosage was concerned.

The extraction yield of the hydroethanolic extracts of the two selected plants was 14% and 16% for* E*.* floribundus *(HEEF) and* A*.* indica *(HEAI), respectively.

### 3.3. Antimalarial Activity

Plant extracts administered at different doses reduced parasitemia in* P. berghei *infected mice in a dose-dependent manner. In fact, at doses of 60, 120, and 240 mg/kg, the hydroethanolic extract of* E*.* floribundus *(HEEF) reduced the parasitemia by 30.5, 57.3, and 62.4% and 39.6, 57.3, and 62.2% for female and male mice, respectively (Table 1).

At the doses of 75, 150, and 300 mg/kg for the hydroethanolic extract of* A*.* indica *(HEAI), there was a reduction of parasitemia by 49.8, 55.2, and 68% and 50.8, 55.9, and 69.3%, respectively, for female and male mice (Table 2).

The most efficient doses of extracts for female and male mice were 120 mg/kg/day for HEEF (57.3 ± 3.8%-57.3 ± 3.9%) and 300 mg/kg/day (68 ± 1.1%-69.3 ± 1.4%) for HEAI. Moreover, no significant difference between the effects of HEEF extract was found at doses 120 and 240 mg/kg (p>0.05). Despite the inhibitory effect of HEAI and HEEF extracts, their activity was far less important compared to that of quinine that showed an activity 70 to 140 times more strongly than the investigated extracts.

### 3.4. Effects of* E*.* floribundus* and* A*.* indica* Extracts on Parasitized Mice Temperature

**Figure 1:**
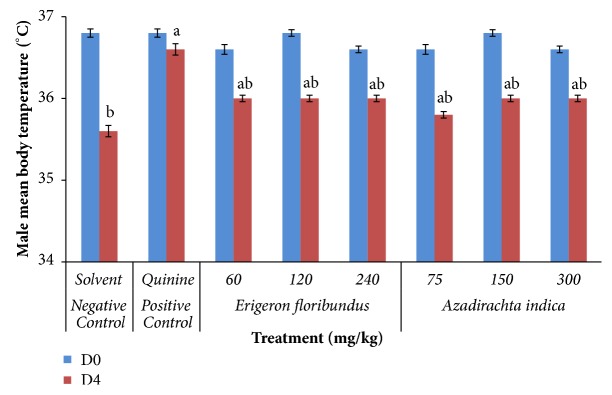
Effects of* E*.* floribundus* and* A*.* indica* hydroethanolic extracts on the temperature of parasitized male mice. D0: day 0; D4: day 4; n = 6; ^a^p < 0.05 compared to negative control at day 4; ^b^p < 0.05 compared to positive control at day 4.

**Figure 2:**
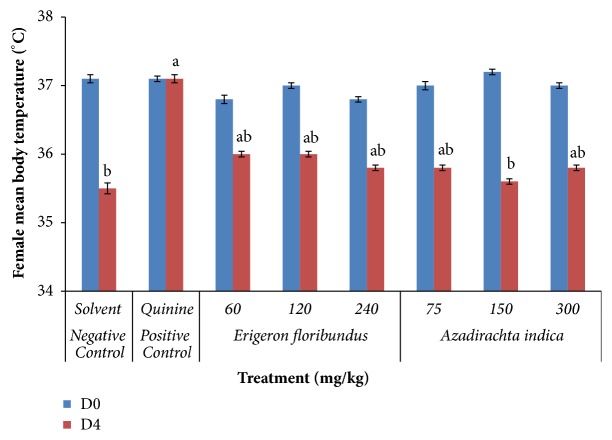
Effects of* E*.* floribundus* and* A*.* indica* hydroethanolic extracts on the temperature of parasitized female mice. D0: day 0; D4: day 4; n = 6; ^a^*p* < 0.05 compared to negative control at day 4; ^b^*p* < 0.05 compared to positive control at day 4.

None of the doses of* E*.* floribundus* and* A*.* indica* extracts significantly improved the body temperature of* P. berghei* infected mice at day 4 compared to day 0 as indicated in Figures 1 and 2.* P. berghei *infected mice treated with hydroethanolic* E*.* floribundus* and* A*.* indica* extracts reduced the body temperature from 36.6-36.8°C for male and 36.8-37.1°C for female at day 0 to 35.8-36°C for male and 35.6-36°C for female on day 4, corresponding to temperature drop of 0.6 to 0.8°C for male and 0.8 to 1.6°C for female mice, respectively. In the control groups, temperature reduction was 1.2°C for male and 1.6°C for female in the negative control and 0.2°C for male and none for female in the positive control. Overall, HEEF and HEAI extracts caused significant (*p*<0.05) but not dose-dependent drop of mice's temperature in the range of 0.4-0.6°C for male (Figure 1) and 0.8-1.2°C for female (Figure 2) when compared to the positive controls, as well as a significant (*p*<0.001) attenuation of the body temperature drop in mice compared to negative control, except for the 150 mg/kg dose in the female group (Figure 2).

### 3.5. Acute Toxicity Study

Over the 14 days period following administration of extracts to* R*.* norvegicus* at doses of 2000 mg/kg and 5000 mg/kg, neither significant behavioural changes, nor morbidity/mortality was recorded among tested animals. The 50% lethal dose (LD_50_) was therefore considered to be higher than 5000 mg/Kg for both plants extracts. Moreover, no animals showed changes in the overall appearance and the somatosensory motricity during the observation period. No manifestation of tremors, convulsions, salivation, diarrhoea, coma, or abnormal behaviours such as self-injury or walking backward was observed for the two extracts. However, sleep events were observed in all animals during the first 30 minutes upon gavage. This sleep was fleeting in controls but was reversible after a few hours in the treated animals (Supplementary Material 2). In addition, the animal body weight did not significantly increase during the test period with a maximum weight gain of 2.6 g for the treated groups compared to 2.7 g in the control group (Table 3).

### 3.6. Effect of Subacute Extracts Administration on the Behaviour and the Relative Body Weight of Rats

Rats were daily administered oral doses of extracts* A*.* indica* at 75, 150, and 300 mg/kg b.w. and* E*.* floribundus* at 60, 120, and 240 mg/kg b.w. for a period of 28 days. Upon treatment, no change was observed in the animals during the whole period of treatment. The rats normally grown over the 28 days period of treatment in the treated and control groups as indicated in Table 4, with a weight gain of approximately 10 to 20 g.

The profiles of the biochemical parameters (ALAT, ASAT, and creatinine) evaluated upon treatment with* E*.* floribundus *and* A*.* indica *extracts indicated no overall significant changes. However, ASAT level increased in the group of animals treated with* A*.* indica *extract at a dose of 300 mg/kg b.w. when compared to the control group (Table 5).

On day 28, an autopsy of the animals revealed a general darkening of the liver with the presence of nodules in animals treated with* A*.* indica* at a dose of 300 mg/kg b.w. and two animals treated with* E*.* floribundus *at doses of 120 mg/kg and 240 mg/kg, respectively, compared to the controls. An examination of the thoracic cavity revealed the presence of nodules in the lungs of two animals, respectively, treated with the extracts of* A*.* indica* at 150 mg/kg b.w. and* E*.* floribundus* at 120 mg/kg b.w. when compared to the negative controls. Also, the kidney of one animal treated with* E*.* floribundus* at 240 mg/kg b.w. was atrophied. Besides, all the organs of the other animals presented no distinctive sign-treatment effects compared to the negative control animals. Nevertheless, no differences were observed in weights of organs,* namely,* lungs, livers, kidneys, hearts, and spleens between treated and control mice (Table 4).

## 4. Discussion

Medicinal plants have shown to be interesting sources of potent antimalarial drugs. Thus, this study examined the* in vivo* potency of* E*.* floribundus* and* A*.* indica* extracts against* P*.* berghei *NK65 strain in infected mice. The results of this study showed that the hydroethanolic extracts of* E*.* floribundus* and* A*.* indica* reduced the parasitemia of* P*.* berghei* in white BALB/c male and female mice in a range of 30-69%. This range of inhibition may be considered as the moderate antimalarial activity as otherwise stated [26]. This result is consistent with data from* in vivo* studies carried out with different extracts of* A*.* indica* [9, 10, 21].

In this preliminary study adopting protocols which simulate the recommendation of traditional healers in terms of dosage and solvents, the comparative antimalarial activity observed between the plants, combined with the safety of* E. floribundus*, supports its traditional use for the management of malaria in the locality of Dibi. This plant may also be used as alternative in the locality as* A. indica* present signs of toxicity at a high dose.

In the oral route, bioactive molecules cross several barriers and enzymatic systems before reaching the systemic circulation, the consequence being the modification of bioactive molecules by metabolism, and thus either improve or reduce the antiplasmodial activity. This suggested that the antiplasmodial activity of metabolically activated compounds can also be observed* in vivo* analysis.

In fact, the antimalarial activity observed could be ascribed to secondary metabolites that are present in* A*.* indica *and* E*.* floribundus* extracts*. A*.* indica *known as neem is a rich source of alkaloids, terpenoids, steroids, tannins, and flavonoids that were previously shown to have antiplasmodial activity [27]. Moreover, gedunin, nimbinine, azadirachtin, and salannin isolated from neem were found to display very high* in vitro* antiplasmodial activity against sensitive and resistant strains of* P*.* falciparum* [28, 29]. Compounds from* E*.* floribundus *also demonstrated interesting activities, including limonene with IC_50_ values between 0.5 and 9.8 *μ*g/mL against* P. falciparum* FcB1 [30, 31]. In addition,* E*.* floribundus* is also rich in flavonoids, saponins, polyphenols, alkaloids, glycosides, and tannins [14]. It is most abundant phenolic compounds named caffeoyl quinic derivatives which displayed moderate to weak (IC_50_ >29 *μ*M) antiplasmodial activity against HB3 and Dd2 strains of* P. falciparum* [32, 33] while luteolin, quercetin, and apigenin demonstrated previously moderate activity of less than 20 *μ*M against 3D7 and 7G8 strains of* P. falciparum* [34]. However, further investigation on metabolites rich fraction especially for the not well known plant* E. floribundus* harvested in the Dibi locality should provide more information regarding the potent antimalarial compounds.

As stated overhead, previous studies on* in vivo *antimalarial activity of* A*.* indica* have also been catalogued to discuss our results. Kingsley et al. (2012) tested the aqueous extract of* A. indica* leaves and got parasite reductions of 48.95% at 50 mg/kg and of 62.50% at 100 mg/kg [21], close to our results. Similarly, Oseni and Akwetey [10] study demonstrated that aqueous and ethanolic extracts of* A. indica* at dose of 50, 100, and 200 mg/kg/day caused chemosuppression of 56.96, 59.89, 69.49% and 56.96, 63.15, 69.60%, respectively, on day four, activities superior to ours. Farahna et al. in 2010 found a parasite reduction of 22.8 % at 300 mg/kg with an ethanolic extract of* A. indica* leaves [9], lower than ours at the same dose although many factors such as extraction solvent and protocol or harvesting period can influence the results. For the second plant* E. floribundus*, this is the first time that its potential antimalarial activity is mentioned in the literature.

In our study, the use of ethanol solvent combined to water at a proportion 70:30 did not increase significantly the activity of the plants compared to studies done using a single solvent [10]. Much remains to be done to identify the best solvent and/or the good proportion to be used for substantial enhancement in the antimalarial activity of* A. indica *and* E. floribundus*. Similarly, studies are scheduled to confirm this activity at higher doses.

Rectal thermometry is a common method of measuring body temperature in rodents to control energy balance or energy metabolism* in vivo*. Anemia, body weight loss, and temperature reductions are common characteristics of* P. berghei* infected mice [35]. A decrease in the metabolic rate of infected mice occurs before death and is accompanied by a corresponding decrease in internal body temperature [36]. So, ideal antimalarial agents obtained from plants are expected to prevent these decreases in infected mice due to the rise in parasitemia. Knowing that the normal temperature of Balb/c mice varies between 36.5°C and 38°C, the hydroethanolic extracts of* E. floribundus* and* A. indica* significantly induced body temperature drop on day 4 compared to quinine, the recommended drug for severe malaria, but also prevented the temperature decrease due to infection compared to negative controls. The same observation was previously reported and confirmed for* Plasmodium* infected mice [37–39].

In order to gain information on extract's toxicity in animals and verify its direct relevance for protecting human or animal health, an acute toxicity test at limit doses of 2000 then 5000 mg/kg b.w. was performed. The results suggested an LD_50_ dose higher than the limit dose 5000 mg/kg classifying our extracts as practically nontoxic and without any risk for human health as reported in the OECD guidelines [24]. This finding is consistent with a reported LD_50_ oral dose of ethanolic extract of* A. indica* of higher than 2000 mg/kg, [40] confirming the clinical safety of the plant [10]. Apart from the study on* Conyza sumatrensis* [41], a synonym of* E. floribundus*, showing no mortality for up to 3000mg/kg in acute toxicity and nonsignificant changes in rats for up to 1000 mg/kg dose in the subchronic study, little information is available on the effect of* E. floribundus* extracts on animals if not the drowsiness and the tiredness observed on rats receiving aqueous extract of the same plant at doses of 1600 and 3200 mg/kg b.w. [15].

A subacute toxicity was also performed in this study to evaluate possible adverse effects of repeated extract's administration to rats, at doses corresponding to those of antimalarial assessment. No significant change was observed in body and organ weights of rats, as well as in biochemical parameters except an increase in serum ASAT at dose of 300 mg/kg/day, suggesting muscular dysfunction or damage to internal organs. In fact, the body weight changes are markers of adverse effects of drugs and chemicals, considered as statistically significant if a body weight loss is of more than 10%. Organ weight also is an important indicator of physiological and pathological status of animals, fundamental to confirm whether the organ was exposed to lesions or not [42]. Indeed, symptoms of neurological or respiratory disorder and mortality were recorded in mice receiving ethanolic extract of neem at doses up to 1000 mg/kg due to* P*.* berghei* infection [9]. Macroscopic exams showed in this study that the target organs of* A. indica* and* E. floribundus* hydroethanolic extracts were lungs, liver, and kidneys at doses between 120 and 300 mg/kg/day. The heart, liver, kidney, spleen, and lungs are the primary organs affected by metabolic reaction caused by toxicant; blood urea nitrogen (BUN) and creatinine, the parameters to evaluate kidney function and transaminases such as ALAT and ASAT, the good indicators of liver function used as biomarkers of drugs toxicity [42].

## 5. Conclusion

At the end of this study,* E*.* floribundus *and* A*.* indica* extracts have reduced the parasitemia in* P. berghei* infected mice in dose-dependent manner, supporting the traditional use of these plant species by Dibi population of Adamawa Region in the management of malaria and related symptoms. The implication of this finding is that hydroethanolic extracts possesses antimalarial effect and may, therefore, serve as potential sources of safe, effective, and affordable antimalarial drugs. Further research focused on toxicity studies coupled with a histological examination of treated animals should provide more information regarding the toxic effects exerted by* A*.* indica* extract on the liver.

## Figures and Tables

**Table 1:** Effects of hydroethanolic extracts of *E*. *floribundus *on parasitemia.

**Treatment**	**Sex**	**Dose (mg/kg)**	**Mean Parasitemia (**%**) ± SD**	**Parasite reduction (**%**)**
**HEEF**	M	60	32.85 ± 2.78 *∗∗∗*	39.57± 5.12^ c^
		120	23.22 ± 2.05 *∗∗∗*	57.28± 3.77^ b^
		240	20.57 ± 1.16 *∗∗∗*	62.16± 2.19^ b^
	F	60	33.08 ± 2.35 *∗∗∗*	30.46± 4.30^ c^
		120	23.37 ± 2.15 *∗∗∗*	57.32± 3.93^ b^
		240	20.61 ± 1.27 *∗∗∗*	62.36± 2.32^ b^
**Quinine**	M	10	0.23 ± 0.16 *∗∗∗*	99.58± 0.29^ a^
	F	10	0.29 ± 0.28 *∗∗∗*	99.47± 0.55^ a^
**Distilled water**	M	200 *µ*L	54.36 ± 3.43	0.00
	F	200 *µ*L	54.76 ± 3.01	0.00

*∗∗∗P *< 0.001, statistically significant difference in comparison with control group (distilled water) using student t-test; n=6. HEEF: hydroethanolic extracts of *E*.* floribundus*. M: male; F: female.

a, b, and c represent comparison between groups. ANOVA followed by Tukey HSD test.

**Table 2:** Effects of hydroethanolic extracts of *A*.* indica *on parasitemia.

**Treatment**	**Sex**	**Dose (mg/kg)**	**Mean parasitemia (**%**) ± SD**	**Parasite reduction (**%**)**
**HEAI**	M	75	26.95 ± 1.80 *∗∗∗*	50.79±3.31^ c^
		150	23.97 ± 0.74 *∗∗∗*	55.91±1.36^ c^
		300	16.70 ± 0.74 *∗∗∗*	69.28±1.36^ b^
	F	75	26.95 ± 1.95 *∗∗∗*	49.75± 3.64^d^
		150	24.04 ± 0.71 *∗∗∗*	55.17± 1.32^ c^
		300	17.16 ± 0.59 *∗∗∗*	68.00± 1.10^ b^
**Quinine**	M	10	0.23 ± 0.16 *∗∗∗*	99.58±0.29^ a^
	F	10	0.25 ± 0.16 *∗∗∗*	99.53± 0.31^ a^
**Distilled water**	M	200 *µ*L	54.36 ± 3.43	0.00
	F	200 *µ*L	53.63 ± 2.56	0.00

*∗∗∗P *< 0.001, statistically significant difference in comparison with control group (distilled water) using Student t-test; n=6. HEAI: hydroethanolic extracts of *A*.* indica. *M: Male; F: female.

a, b, and c represent comparison between groups. ANOVA followed by Tukey HSD test.

**Table 3:** Body weights of mice subjected to acute toxicity assessment of the hydroethanolic extracts of *E*. *floribundus* and *A*.* indica.*

**Groups**	**Body weight (g) ± SD**		
	**Day 0**	**Day 7**	**Day 14**
***(A) Mice treated with HEEF***			
**Control**	22.0 ± 0.7	23.7 ± 0.9	24.8 ±0.6
**2000 mg/kg**	23.3 ± 0.9	24.0 ± 0.7	24.9 ± 0.5
**5000 mg/kg**	22.1 ± 0.3	23.2 ± 0.7	24.1 ±0.2
***(B) Mice treated with HEAI***			
**Control**	22.0 ± 0.7	23.7 ± 0.9	24.8 ± 0.6
**2000 mg/kg**	22.1 ± 0.7	23.3 ± 0.3	24.6 ± 0.3
**5000 mg/kg**	22.0 ± 0.6	22.5 ± 1.0	24.7 ± 0.8

Female mice were administered a single dose of each plant extract at doses of 2000 mg/kg (5) and 5000 mg/kg (3) and mean body weight was recorded before the treatment (day 0) and on days 7 and 14. The control group received 200 *µ*L of distilled water. HEEF; hydroethanolic extracts of *E*.* floribundus*; HEAI: hydroethanolic extracts of *A*.* indica.*

**Table 4:** Body and organ weights of rats subjected to subacute toxicity assessment of the hydroethanolic extracts of *E*. *floribundus* and *A*.* indica.*

**Groups**	**Body weight (g) ± SD**				
	**Day 0**	**Day 7**	**Day 14**	**Day 21**	**Day 28**
***(A) Rats treated with HEEF***					
Control	123.3±9.1	127.8±8.8	131.8±9.2	136.8±8.8	143.5±9.5
60 mg/kg	123.3 ± 10.3	126.3 ± 9.7	129.8 ± 9.6	131.8 ± 9.9	134.7±10.2
120 mg/kg	123.3 ± 8.8	126.3 ± 9.0	129.7 ± 11.5	130.3 ± 8.2	133.0±7.7
240 mg/kg	123.0 ± 13.1	126.4 ± 12.5	130.4 ± 8.6	132.0±10.7	134.2±10.6

**Groups**	**Organ weight (g) ± SD**				
	**Liver**	**Heart**	**Kidneys**	**Lungs**	**Spleen**

Control	5.72 ± 0.66	0.62 ± 0.05	1.28 ± 0.13	1.16 ± 0.11	0.78 ± 0.19
60 mg/kg	5.15 ± 0.58	0.60 ± 0.08	1.26 ± 0.13	1.51 ± 0.65	0.80 ± 0.14
120 mg/kg	4.54 ± 1.46	0.54 ± 0.05	1.21 ± 0.09	1.39 ± 0.36	0.70 ± 0.15
240 mg/kg	5.16 ± 0.32	0.52 ± 0.06	1.17 ± 0.04	1.40 ± 0.53	0.79 ± 0.19

**Groups**	**Body weight (g) ± SD**				
	**Day 0**	**Day 7**	**Day 14**	**Day 21**	**Day 28**

***(B) Rats treated with HEAI***					
Control	123.3 ± 9.11	127.8 ± 8.8	131.8 ± 9.2	136.8 ± 8.8	143.5 ± 9.5
75 mg/kg	123.2 ± 15.3	127.0 ± 15.0	130.0 ± 14.5	136.6 ± 13.7	139.3± 4.6
150 mg/kg	123.5 ± 10.2	128.0 ± 10.4	131.5 ± 9.4	136.0 ± 9.3	141.0±10.0
300 mg/kg	122.7 ± 5.8	126.5 ± 5.5	130.0 ± 5.8	133.7 ± 6.3	137.2 ± 6.5

**Groups**	**Organ weight (g) ± SD**				
	**Liver**	**Heart**	**Kidneys**	**Lungs**	**Spleen**

Control	5.72 ± 0.66	0.62 ± 0.05	1.28 ± 0.13	1.16 ± 0.11	0.78 ± 0.19
75 mg/kg	5.65 ± 0.58	0.56 ± 0.06	1.25 ± 0.06	1.09 ± 0.11	0.81 ± 0.13
150 mg/kg	5.93 ± 0.75	0.55 ± 0.05	1.33 ± 0.10	1.25 ± 0.37	0.74 ± 0.11
300 mg/kg	5.08 ± 0.37	0.54 ± 0.04	1.22 ± 0.06	1.37 ± 0.25	0.86 ± 0.44

Mice (5) were administered with 60, 120, and 240mg/kg doses of HEEF, with 75, 150, and 300mg/kg doses of HEAI for 28 days, and mean body weight and organ weight were recorded before the treatment (day 0) and on days 7, 14, 21, and 28. The control group received 200 *µ*L of distilled water.

**Table 5:** Effects of *E*.* floribundus* and *A*.* indica* hydroethanolic extracts on biochemical parameters.

**Dose (mg/kg)**	**Parameter**		
	**ALAT (U/L)**	**ASAT (U/L)**	**Creatinine (mg/L)**
***(A) Mice treated with HEEF***			
**Control**	55.00±6.1^a^	153.85±18.5^a^	4.83±0.4^a^
**60**	54.67±10.8^a^	181.45±22.6^a^	4.72±0.3^a^
**120**	53.33±7.0^a^	162.35±11.5^a^	4.57±0.2^a^
**240**	49.00±11.9^a^	175.08±36.6^a^	4.88±0.6^a^
***(B) Mice treated with HEAI***			
**Control**	55.00±6.1^a^	153.85±18.5^a^	4.83±0.4^a^
**75**	64.67±17.4^a^	203.93±42.1^a^	4.50±0.7^a^
**150**	61.33±17.4^a^	191.38±36.3^a^	4.30±0.5^a^
**300**	58.66±12.7^a^	213.58±30.5^b^	4.55±0.2^a^

a and b represent comparison between groups. ANOVA followed by Tukey HSD test. n=6. HEEF: hydroethanolic extracts of *E*.* floribundus*; HEAI: hydroethanolic extracts of *A*.* indica.*

## Data Availability

The data used to support the findings of this study are included within the article.

## Supplementary Materials

Supplementary Materials Table S1: plants used in the treatment of malaria in Dibbi locality. Table S2: behavioural observations of acute toxicity study with* Erigeron floribundus* and* Azadirachta indica*.
