# Supplementary material for: Hydroethanolic Extracts of Erigeron floribundus and Azadirachta indica Reduced Plasmodium berghei Parasitemia in Balb/c Mice
Source: Evid Based Complement Alternat Med. 2018 Oct 21;2018:5156710. doi: 10.1155/2018/5156710 (PMC6215572; doi:10.1155/2018/5156710)
Supplement: Supplementary Materials — Table S1: plants used in the treatment of malaria in Dibbi locality. Table S2: behavioural observations of acute toxicity study with Erigeron floribundus and Azadirachta indica. [file 5156710.f1.pdf]

SUPPLEMENTARY MATERIALS

1- Plants used in the treatment of malaria in Dibbi locality

Family	Plant name	Local name (fufuldé)	Part use	Growth form	Mode of preparation	Number of respondents	Other diseases treated
Asteraceae	*Bidens pilosa* Linn. Var. Radita	Nyakamre	leaves	Herb	Maceration	5	Hypertension, Typhoid
Asteraceae	*Erigeron floribundus* (Kunth) Sch.Bip.	Guéné hadhi	leaves	Herb	Maceration	2	Typhoid
Verbenaceae	*Lantana camara* L.	/	leaves	shrub	Decoction	5	Typhoid
Amaranthaceae	*Achyranthes aspera* L.	Saille doho	leaves	herb	Maceration/ Decoction	2	Nerve sickness
Meliaceae	*Azadirachta indica* A. Juss	Gagne	leaves, fruit	tree	Decoction	15	Yellow fever, AIDS, abdominal pains, hepatitis
Caesalpiniaceae	*Tamarindus indica*	Djabbe	leaves, bark	tree	Decoction	12	Filariasis
Caesalpiniaceae	*Senna* occidentalis L.	Kaccu-kaccunga	leaves, seed	shrub	Decoction	10	Typhoid, yellow fever, abdominal pains, hepatitis
Rubiaceae	*Spermacoce stachydea* de C.	/	leaves, root	herb	Decoction	2	Analgesic
Liliaceae	*Aloe verra* (L.) Burm.F.	/	leaves	herb	Maceration	2	Helminthiasis, dermatosis, typhoid
Caricaceae	*Carica papaya* L.	Dukudjee	leaves, root	tree	Maceration/ Decoction	10	Typhoid, tooth decay
Myrtaceae	*Eucalyptus sp*	/	leaves, bark	tree	Decoction	8	Typhoid

2- Behavioural observations of acute toxicity study with *Erigeron floribundus* and *Azadirachta indica*

Dose (mg/kg)	Time of observation					
	**0 - 30 min**	**1/2 - 2 h**	**2 - 4 h**	**4 - 24 h**	**24 - 48 h**	**2 - 14 j**
(A) Mice treated with HEEF						
**Control**	sleep	RAS	RAS	RAS	RAS	RAS
**2000**	sleep	sleep	sleep	RAS	RAS	RAS
**5000**	sleep	sleep	sleep	RAS	RAS	RAS
(B) Mice treated with HEAI						
**Control**	sleep	RAS	RAS	RAS	RAS	RAS
**2000**	sleep	sleep	RAS	RAS	RAS	RAS
**5000**	sleep	sleep	RAS	RAS	RAS	RAS

RAS = Nothing to report

HEAI : hydroethanolic extracts of *Azadirachta indica*; HEEF : hydroethanolic extracts of *Erigeron floribundus*
